# Nutritional quality, mineral and antioxidant content in lettuce affected by interaction of light intensity and nutrient solution concentration

**DOI:** 10.1038/s41598-020-59574-3

**Published:** 2020-02-18

**Authors:** Jiali Song, Hui Huang, Yanwei Hao, Shiwei Song, Yiting Zhang, Wei Su, Houcheng Liu

**Affiliations:** College of Horticulture, South China Agricultural University, Guangzhou, 510642 China

**Keywords:** Physiology, Plant sciences

## Abstract

Light and nutrient are important factors for vegetable production in plant factory or greenhouse. The total 12 treatments which contained the combination of four light intensity (150, 250, 350 and 450 μmol · m^−2^ · s^−1^) and three nutrient solution concentration (NSC) (1/4, 1/2, 3/4 strength NSC) were established for investigation of lettuce growth and quality in a growth chamber. The combination of light intensity and NSC exhibited significant effects on photosynthetic pigment, nutritional quality, mineral content and antioxidant capacity. That a higher light intensity were readily accessible to higher chlorophyll a/b showed in lettuce of treatment of 350 μmol · m^−2^ · s^−1^ × 3/4NSC and 450 μmol · m^−2^ · s^−1^ × 1/4NSC. Lower total N contents, higher content of soluble protein, vitamin C, soluble sugar and free amino acid exhibited in lettuce under treatment of 250 and 350 μmol · m^−2^ · s^−1^ × 1/4NSC or 3/4NSC. With increasing NSC and LED irradiance, the content of total P and K in lettuce increased and decreased, respectively. The highest and lowest total Ca content were found in treatment of 150 μmol · m^−2^ · s^−1^ × 1/4NSC and 450 μmol · m^−2^ · s^−1^ × 1/4NSC, respectively, and higher content of total Mg and Zn was observed under 250 μmol m^−2^ s^−1^ × 1/4NSC and 150 μmol · m^−2^ · s^−1^ × 3/4NSC, respectively. The antioxidant contents generally decreased with increasing NSC level. The higher antioxidant content and capacity occurred in lettuce of 350 μmol · m^−2^ · s^−1^ × 1/4NSC treatment. The interaction of 350 μmol · m^−2^ · s^−1^ × 1/4NSC might be the optimal condition for lettuce growth in plant factory.

## Introduction

Lettuce (*Lactuca sativa* L.) is one of the most important vegetable in the world, which is main crop in plant factory. The secondary metabolite in vegetable plays key roles for human body health, such as phenolic compound, vitamin A and C, and carotenoid. These compounds have a function on nutrition and health care^1^, which could enhance anti-oxidation ability of human body, and the suppression of inflammatory disease and cancer^2,3^. Nutritional quality of lettuce is affected by light and nutrient solution in plant factory. Light (including light quality, intensity and photoperiod) plays a crucial role in improvement of plant nutrition quality^4,5^. The proper ratio of red and blue light is essential for plant growth and development^6^. Light intensity not only positively regulates lettuce biomass and morphology, but also nutrition quality and activities of anti-oxidative enzymes^7,8^. The content of soluble sugars and ascorbic acid in lettuce increases with increasing light intensity^9^. Different light intensity is required by different plant for nutritional quality and growth. The highest content of lutein, ß-carotene and chlorophyll in leave shows at 335 μmol · m^−2^ · s^−1^ for kale, but 200 μmol · m^−2^ · s^−1^ for spinach^10^. In ten leafy vegetables (chicory, green lettuce, lamb’s lettuce, mizuna, red chard, red lettuce, rocket, spinach, swiss chard, and tatsoi), higher leaf dry matter, content of protein, K, Ca and Mg, hydrophilic antioxidant activity, and lipophilic antioxidant activity are observed under low light intensity than high intensity^11^.

In hydroponic systems, the arrangement of nutrient solution could modify the quality and yield of vegetable. Nitrate content in lettuce is closely related to solution nitrate concentration^12^. Total soluble solids concentration in cherry tomato exhibits the highest in the continuous high EC treatment, and the lowest in the continuous low EC treatment^13^. Growth and quality of hydroponic plant especially in closed plant factory is affected by integrated factors, such as light, nutrient supply, CO_2_ concentration. It had been demonstrated that microalgae (*Scenedesmus* sp.) exhibits the highest weight recovery efficiency in combination of low light intensity and nutrient supply condition^14^. The combination of high light illumination and low nitrogen contributes to increased vitamin C content and reduced nitrate content^15^. High CO_2_ nutrient supply and monochromatic LED synergistically enhance both the lettuce biomass and some amino acids content^16^. However, it is not yet available research which the interaction between light intensity and nutrient solution regulates vegetable growth and phytonutrients.

In this study, the changes of mineral element contents, nutritional quality, antioxidant activity of lettuce in response to different interaction of nutrient solution concentration (1/4, 1/2, and 3/4 strength nutrient solution concentration) and light intensity (150–450 μmol · m^−2^ · s^−1^) based on red and blue LED light were investigated. This is aim to provide valuable insights into the optimal combination of light intensity and nutrient solution to improve quality and yield of lettuce in plant factory.

## Methodology

### Plant materials and growing conditions

Lettuce (*Lactuca sativa* L. cv. Italy) seeds were sowed in sponge block with 1/4 strength nutrient solution concentration (NSC). The full-strength NSC were composed of the following elements: 210.0 mg · L^−1^ N, 31.0 mg · L^−1^ P, 234.0 mg · L^−1^ K, 160.0 mg · L^−1^ Ca, 48.0 mg · L^−1^ Mg, 64.0 mg · L^−1^ S, 5.6 mg · L^−1^ Fe, 0.5 mg · L^−1^ B, 0.5 mg · L^−1^ Mn, 0.05 mg · L^−1^ Zn, 0.02 mg · L^−1^ Cu, 0.01 mg · L^−1^ Mo. The experiment was performed in a growth chamber in South China Agricultural University and the seedlings with three expended true leaves were transplanted in hydroponic system as follow: 22–25/14–18 °C (day/night), 60–80% relative humidity, the nutrient solution aeration rate of 15 min/h, the nutrient solution was renewed every 10 days. Three light intensity (Photosynthetic Photon Flux Density, PPFD) including 150, 250 and 350 μmol · m^−2^ · s^−1^, basing on LED: red (660 ± 10 nm): blue (460 ± 10 nm) = 2:1 (Fig. S1) and three strength of nutrient solution concentration (1/4. 1/2, 3/4 NSC) (Table S1) were carried out in this study. Therefore, there were 12 treatments of the light intensity interacted with nutrient solution concentration (Table 1).

**Table 1:** 12 treatments of light intensity interacted with nutrient solution concentration.

NSC	1/4	1/2	3/4
Light intensity/μmol · m^−2^ · s^−1^			
150 (red light, 100; blue light, 50)	150 × 1/4	150 × 1/2	150 × 3/4
250 (red light, 167; blue light, 83)	250 × 1/4	250 × 1/2	250 × 3/4
350 (red light, 233; blue light, 117)	350 × 1/4	350 × 1/2	350 × 3/4
450 (red light, 300; blue light, 150)	450 × 1/4	450 × 1/2	450 × 3/4

### The measurement of lettuce growth

At 30 days after transplant, the lettuces were harvested. The fresh weight and dry weight were examined using electronic balance, for dry weight deactivated at 105 °C, then dried at 75 °C for 48 h.

### Pigment content determination

Pigment content was measured colorimetrically according to Gratani^17^. 0.5 g fresh levels dipped in 25 mL acetone alcohol mixture (acetone: alcohol = 1:1) until to turn white to extract chlorophyll a, b and carotenoid. The absorbance of extract liquor was determined by UV-1200 spectrophotometer (Shimadzu, Japan) at 645 nm (OD_645_), 663 nm (OD_663_) and 440 nm (OD_440_). The content of pigment was calculated as follow: chlorophyll a concentration (mg/L) = 12.7 × OD_663_ −2.69 × OD_645_; chlorophyll b concentration (mg/L) = 22.9 × OD_645_ −4.86 × OD_663_; total chlorophyll concentration (mg/L) = 8.02 × OD_663_ + 20.20 × OD_645_; carotenoid concentration (mg/L) = 4.7 × OD_440_ −0.27 × total chlorophyll concentration; chlorophyll a content (mg/g) = (chlorophyll a × 25 mL)/0.5 g; chlorophyll b content (mg/g) = (chlorophyll b × 25 mL)/0.5 g; carotenoid content (mg/g) = (carotenoid × 25 mL)/0.5 g.

### Phytochemical determination

Soluble protein content in lettuce was examined by Coomassie brilliant blue G-250 dye method^18^. A total of 0.5 g fresh lettuces was ground into pulp by liquid nitrogen with 5 mL distilled water. The extract solution was centrifuged at 10000 rpm for 10 min at 4 °C, and 0.05 mL supernatant was combined with 0.95 mL distilled water and 5 mL Coomassie brilliant blue G-250 solution (Sigma, USA, 0.1 g · L^−1^). After 2 min, the soluble protein content was detected at 595 nm by UV-spectrophotometer (Shimadzu UV-16A, Shimadzu, Japan).

Nitrate content was determined by ultraviolet spectrophotometry method^19^. 1 g fresh lettuces were heated on the boiling water bath with 10 mL distilled water for 30 min. After the solution cooling, the extracting solution was filtered by volumetric flask. 0.1 mL sample solution was mixed with 0.4 mL 5% salicylic and sulfuric acid and 9.5 mL 8% NaOH. The nitrated content of mixed solution was determined by UV-VIS spectrophotometer (Shimadzu UV-16A, Shimadzu, Japan) at 410 nm.

Vitamin C was performed by using the 2, 6-dichlorophenol indophenol titration method^20^. 0.5 g fresh leaves were ground into pulp with 3 mL 1% oxalic acid, 1 mL 30% zinc sulfate and 1 mL 15% potassium ferrocyanide. 10 mL extracting solution was mixed with 1 mL phosphate-acetic acid, 2 mL 5% vitriol and 4 mL ammonium molybdate. After 15 min, the mixed solution was determined at 500 nm by UV-VIS spectrophotometer (Shimadzu UV-16A, Shimadzu, Japan).

Soluble sugar content was performed by anthronesulfuric acid colorimetry method^21^. 0.5 g fresh leaves were heated on boiling water bath with 10 mL distilled water for 30 min. 0.1 mL supernatant was mixed with 1.9 mL distilled water, 0.5 mL anthrone ethyl acetate and 5 mL vitriol. After shaking, the soluble sugar was detected by UV-VIS spectrophotometer (Shimadzu UV-16A, Shimadzu, Japan) at 630 nm.

Free amino acid content was determined colorimetrically^19^. 1 g fresh leaves were ground into pulp with 10 mL deionized water, which were heated on a water bath at 80 °C for 30 min. The extract solution was centrifuged at 13000 g for 10 min. The 0.2 mL supernatant was mixed with 0.8 mL 5% (w/v) salicylic acid (Sigma, USA) and 19 mL 4 mol·L^–1^ NaOH. The nitrate content was determined by UV-VIS spectrophotometer (Shimadzu UV-16A, Shimadzu, Japan) at 410 nm.

### Antioxidant component content and capacity determination

Anthocyanin content was determined by spectrometric method^22^. 1.0 g fresh lettuces exacted by 20 mL 60% alcohol (pH = 3.0) were heated on the boiling water bath for 2 h. The exacting solution was filled in volumetric flask. The certain volume of exacting solution using the same extractant to dilute was determined by UV-VIS spectrophotometer (Shimadzu UV-16A, Shimadzu, Japan) at 535 nm.

Polyphenol content was determined by Folin-Cioealteu method^23^. 1 g fresh leaves were pulverized with liquid nitrogen. The sample powder was heated on boiling bath with 8 mL 80% methanol for 60 min, and then the exacting solution was centrifuged with 12000 rpm for 10 min. The supernatant was moved to evaporation flask at 40 °C with 3–4 rpm for 5–6 min. 10 mL distilled water was added to the exacting solution with putting into centrifuge with 8000 rpm for 20 min. 1 mL supernatant was mixed with 7 ml distilled water, 0.5 mL foline-phenol and 11.5 mL 26.7% sodium carbonate. The absorbance of mixed solution was measured at 760 nm by spectrophotometer (Shimadzu UV-16A, Shimadzu, Japan).

Flavonoid content was determined according to Jia *et al*.^24^. The extracted method of flavonoid identified with polyphenol. 1 mL extract solution was added to 11.5 mL 30% alcohol and 0.7 mL 5% NaNO_2_. After 5 min, the reaction solution was mixed with 0.7 mL 10% Al(NO_3_)_3_, and then 6 min later, the mixture was added 5 mL 5% NaOH. Finally, 10 min later, the mixed solution was determined by spectrophotometer (Shimadzu UV-16A, Shimadzu, Japan) at 510 nm.

The 2, 2-diphenyl-1-picrylhydrazyl (DPPH) radical scavenging rate was performed by basing on Tadolini *et al*.^23^. 0.5 g sample solution which identified with exacting polyphenol was added 2.5 mL 65 μmol · L^−1^ DPPH solution. After 30 min, the mixed solution was determined by spectrophotometer (Shimadzu UV-16A, Shimadzu, Japan) at 517 nm.

The value of ferric-reducing antioxidant power (FRAP) was performed according to Benzie and Strain^25^. 0.4 mL sample solution which identified with exacting polyphenol was mixed with 3.6 mL mixed solution (0.3 mol · L^−1^ acetate buffer; 10 mmol · L^−1^ TPTZ; 20 mmol · L^−1^ FeCl_3_ = 10:1:1) for 10 min at 37 °C. The mixed solution was determined by spectrophotometer (Shimadzu UV-16A, Shimadzu, Japan) at 593 nm.

### Mineral element determination

Fresh lettuce was heated to de-enzyme at 105 °C for 1 h, then dried at 75 °C. The kiln-dried sample was smashed and stored to measure mineral element content. Total N, P and K was determined by using Ojeda’s^26^, Mo-Sb Colorimetry^27^, and flame photometry method^28^, respectively, while Ca, Mg and Zn content were performed by using atomic absorption spectrophotometry method^29^.

### Statistical analysis

All the assays were analyzed in triplicates.Variance analysis of one-way in single light intensity or NSC factor, and two-way in the combination of light intensity and NSC was performed by using SPSS17.0 software to determine the significance at *p* ≤ 0.05 and *p* ≤ 0.01 level.

## Results and Analysis

### Growth and weight

Light intensity, NSC and their interaction exhibited a prominent difference in lettuce leaf number, fresh weight (FW) and dry weight (DW) of plant and shoot (Table 2 and Fig. S2). There was the maximum leaf number in 1/2 and 3/4NSC under 350 μmol · m^−2^ · s^−1^ treatment. A tendency which plant and shoot weight increased at first then decreased with increasing light intensity was observed. Lettuce fresh and dry weight under the combination of 350 μmol · m^−2^ · s^−1^ × 3/4NSC was the highest (Table 2). These results implied that 350 μmol · m^−2^ · s^−1^ × 3/4NSC was the suitable condition for lettuce growth.

**Table 2:** Growth lettuce affected by different light intensity × NSC.

Treatments		Leaf number	Weight (g per plant)			
Light intensity (μmol · m^−2^s^−1^)	Nutrient solution concentration		Plant FW	Shoot FW	Plant DW	Shoot DW
150	1/4	14.33 ± 0.667ef	44.77 ± 2.832e	42.00 ± 2.397e	1.77 ± 0.088 f	1.53 ± 0.088 f
	1/2	13.33 ± 0.333 f	54.57 ± 3.717de	51.57 ± 3.661de	2.40 ± 0.153e	2.17 ± 0.120d
	3/4	16.00 ± 0.00bcde	59.03 ± 1.670d	55.63 ± 1.670d	2.33 ± 0.088e	2.13 ± 0.088d
250	1/4	15.33 ± 0.667cdef	73.70 ± 1.473bc	68.57 ± 1.671bc	3.13 ± 0.033d	2.90 ± 0.000c
	1/2	14.67 ± 0.333def	65.37 ± 3.002 cd	61.83 ± 2.649 cd	3.37 ± 0.273d	2.93 ± 0.186c
	3/4	17.33 ± 1.202abc	100.67 ± 2.293a	94.30 ± 2.381a	4.00 ± 0.265bc	3.63 ± 0.233ab
350	1/4	17.00 ± 0.577bc	79.40 ± 5.059b	73.63 ± 4.589b	3.53 ± 0.233 cd	3.13 ± 0.176c
	1/2	19.33 ± 0.667a	84.30 ± 6.178b	77.30 ± 6.564b	4.10 ± 0.208ab	3.60 ± 0.208b
	3/4	19.33 ± 0.333a	103.43 ± 4.853a	95.03 ± 4.133a	4.60 ± 0.100a	4.10 ± 0.058a
450	1/4	17.67 ± 0.667ab	75.73 ± 4.083bc	66.73 ± 4.326bc	4.13 ± 0.203ab	3.63 ± 0.203ab
	1/2	17.67 ± 1.202ab	98.60 ± 1.054a	89.73 ± 0.203a	4.33 ± 0.033ab	3.90 ± 0.058ab
	3/4	16.67 ± 0.333bcd	81.17 ± 2.674b	72.77 ± 2.088b	4.10 ± 0.153ab	3.60 ± 0.153b
ANOVA (F value)	Light intensity	20.299**	63.098**	52.256**	86.704**	89.529**
	NSC	4.061*	24.697**	23.716**	13.202**	14.84**
	Light intensity × NSC	2.973*	9.972**	9.471**	2.564*	3.513*

FW = fresh weight, DW = dry weight. The results showed by mean ± standard error. Different letters mark in tables indicated significant difference (P ≤ 0.05, Tukey’s test). * and ** represented the significant difference at *p* ≤ 0.05 and *p* ≤ 0.01, respectively. Significant differences among the treatments were determined by SPSS 17.0 for ANOVA.

### Photosynthetic pigment content

As shown in Table 3, light intensity, NSC and their combinations significantly modulated content of chlorophyll a, chlorophyll b, and the ratio of chlorophyll a to b (chlorophyll a/b). However, there was no significant difference in carotenoid content among all treatments. The highest chlorophyll a content was found under 350 μmol · m^−2^ · s^−1^ × 1/2NSC, whereas the lowest under 450 μmol · m^−2^ · s^−1^ × 1/2NSC. Chlorophyll b content descended with light intensity increasing. A lower chlorophyll a/b was observed under low light intensity treatments (150 and 250 μmol · m^−2^ · s^−1^), but no difference under different NSC. Chlorophyll a/b significantly improved with increasing NSC under 350 μmol · m^−2^ · s^−1^, along with the highest at 3/4NSC. Hence, 150 μmol · m^−2^ · s^−1^ × 1/4NSC was beneficial to accumulation of chlorophyll a and b, and total chlorophyll, while the 350 μmol · m^−2^ · s^−1^ × 3/4NSC treatment contributed to the promotion of chlorophyll a/b.

**Table 3:** Photosynthetic pigment level affected by light intensity × NSC.

Treatments		Photosynthetic pigment content (mg/g)				Chlorophyll a/b
Light intensity (μmol · m^−2^ · s^−1^)	Nutrient solution concentration	Chlorophyll a	Chlorophyll b	Total Chlorophyll	Carotenoid	
150	1/4	0.38 ± 0.022abc	0.138 ± 0.0088a	0.525 ± 0.0308a	0.087 ± 0.0087bc	2.76 ± 0.048e
	1/2	0.38 ± 0.022abc	0.130 ± 0.0070ab	0.512 ± 0.0295a	0.085 ± 0.0060bc	2.91 ± 0.020e
	3/4	0.36 ± 0.016bcd	0.127 ± 0.0136ab	0.497 ± 0.0163a	0.079 ± 0.0054c	2.94 ± 0.391e
250	1/4	0.39 ± 0.014abc	0.113 ± 0.0081b	0.507 ± 0.0149a	0.105 ± 0.0055abc	3.46 ± 0.277e
	1/2	0.32 ± 0.010de	0.082 ± 0.0029c	0.405 ± 0.0124bc	0.081 ± 0.0038c	3.87 ± 0.092e
	3/4	0.40 ± 0.012ab	0.129 ± 0.0037ab	0.537 ± 0.0153a	0.097 ± 0.0034abc	3.11 ± 0.018e
350	1/4	0.34 ± 0.027 cd	0.086 ± 0.0048c	0.434 ± 0.0318b	0.089 ± 0.0081abc	3.95 ± 0.086e
	1/2	0.41 ± 0.005a	0.079 ± 0.0053c	0.499 ± 0.0076a	0.116 ± 0.0012a	5.28 ± 0.386de
	3/4	0.28 ± 0.006ef	0.006 ± 0.0004e	0.294 ± 0.0066d	0.084 ± 0.0086bc	50.44 ± 2.205a
450	1/4	0.35 ± 0.005 cd	0.010 ± 0.0005e	0.368 ± 0.0057c	0.110 ± 0.0124ab	36.95 ± 1.714b
	1/2	0.25 ± 0.001 f	0.016 ± 0.0003e	0.268 ± 0.0010d	0.090 ± 0.0008abc	15.26 ± 0.241c
	3/4	0.35 ± 0.008 cd	0.051 ± 0.0011d	0.410 ± 0.0088bc	0.099 ± 0.0187abc	6.87 ± 0.048d
ANOVA (F value)	Light intensity	9.358**	184.564**	49.715**	2.112	401.694**
	NSC	3.616*	3.127	4.459*	0.946	118.356**
	Light intensity × NSC	14.9**	26.497**	20.264**	2.383	416.712**

The results showed by mean ± standard error. Different letters mark in all figures indicated significant difference (P ≤ 0.05, Tukey’s test). * and ** represented the significant difference at *p* ≤ 0.05 and *p* ≤ 0.01, respectively. Significant differences among the treatments were determined by SPSS 17.0 for ANOVA.

### The contents of soluble protein and sugar, vitamin c, nitrate, and free acid

**Figure 1:**
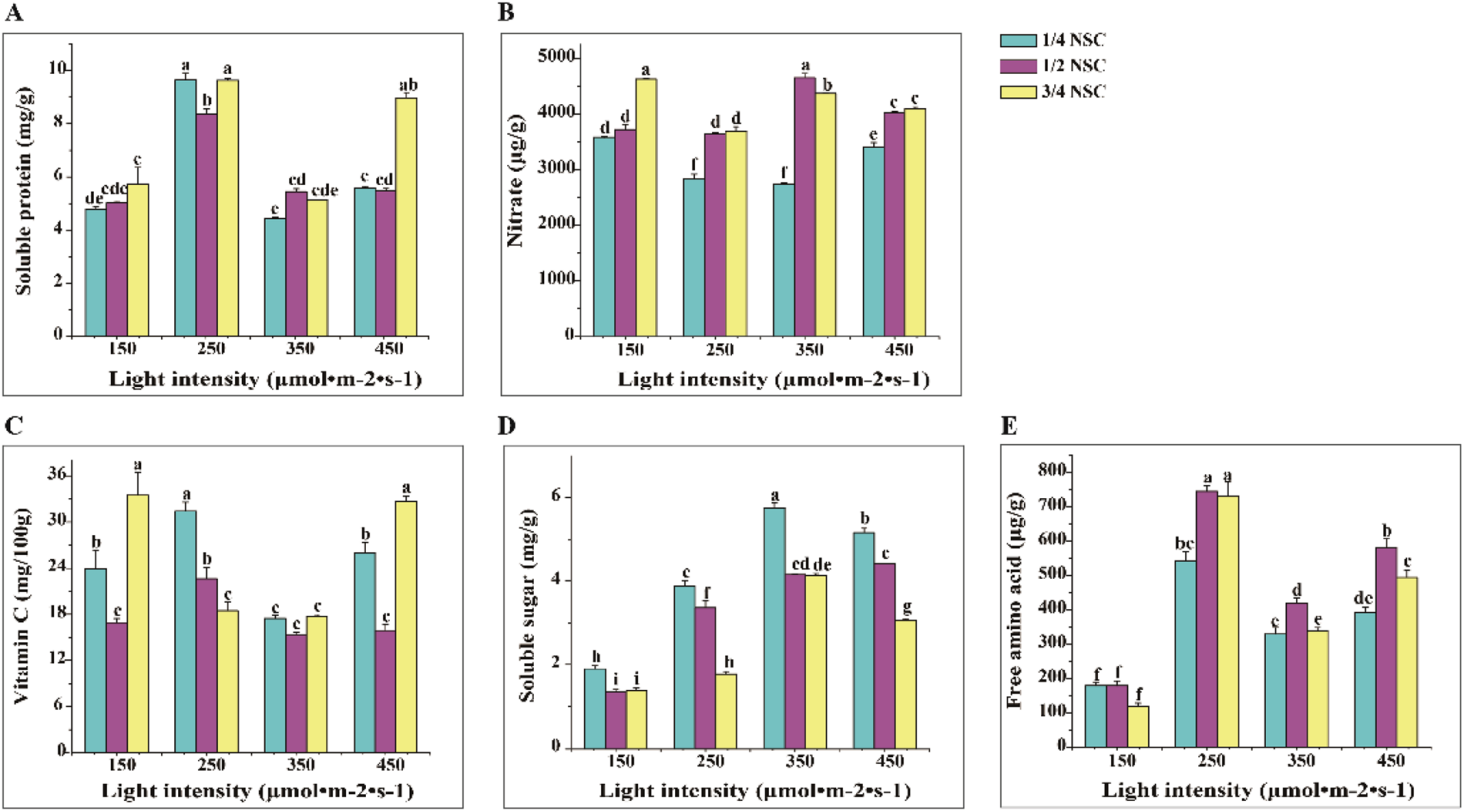
Soluble protein (**A**), nitrate (**B**), vitamin C (**C**), soluble sugar (**D**) and free amino acid (**E**) content regulated by different light intensity × NSC. Different letters mark in all figures indicated significant difference (P ≤ 0.05, Tukey’s test). Significant differences among the treatments were determined by SPSS 17.0 for ANOVA.

Soluble protein content in lettuce was markedly affected by different light intensity (*p* ≤ 0.01), NSC (*p* ≤ 0.01) and their interaction (*p* ≤ 0.01) (Table S2). There was the highest soluble protein content in lettuce under 250 μmol · m^−2^ · s^−1^ × 1/4NSC or × 3/4NSC and next in 450 μmol · m^−2^ · s^−1^ × 3/4NSC treatment (Fig. 1A). These generally showed that soluble protein accumulated more in lettuce at the highest NSC (Fig. 1A).

As shown in Fig. 1B, a positive tendency on nitrate content with increasing NSC was observed. The lowest nitrate content was achieved under 250 or 350 μmol · m^−2^ · s^−1^ × 1/4NSC. Two-way ANOVA analysis confirmed that nitrate content in lettuce was strongly associated with light intensity (*p* ≤ 0.01), NSC (*p* ≤ 0.01) and their interaction (*p* ≤ 0.01) (Table S2).

The light intensity (*p* ≤ 0.01), NSC level (*p* ≤ 0.01) and their combination (*p* ≤ 0.01) exhibited a significant difference on vitamin C content (Table S2). A trend which the content of vitamin C decreased at first then increased with increasing of NSC at 150, 350 and 450 μmol · m^−2^ · s^−1^ was showed, whereas vitamin C content reduced with increasing NSC under 250 μmol · m^−2^ · s^−1^ (Fig. 1C).

Soluble sugar content in lettuce was significantly responsive to light intensity (*p* ≤ 0.01), NSC (*p* ≤ 0.01) and their interaction (*p* ≤ 0.01) (Table S2). It increased at irradiance from 150 μmol · m^−2^ · s^−1^ to 350 μmol · m^−2^ · s^−1^ then decreased at 450 μmol · m^−2^ · s^−1^, while deceased with increasing NSC level, with the highest under 350 μmo · m^−2^ · s^−1^ × 1/4NSC (Fig. 1D).

Light intensity (*p* ≤ 0.01), NSC (*p* ≤ 0.01) and their combination (*p* ≤ 0.01) significantly affected the content of free amino acid in lettuce (Table S2). The content of free amino acid in lettuce under 250 μmol · m^−2^ · s^−1^ × 1/2NSC or × 3/4NSC dramatically exceed than other irradiance conditions, with peaking at 250 μmol · m^−2^ · s^−1^ × 1/2NSC or × 3/4NSC treatments(Fig. 1E).

In general, 250 μmol · m^−2^ · s^−1^ × 1/4NSC or 3/4NSC contributed to accumulation of vitamin C, soluble protein and free amino acid, and reduction of nitrate content in lettuce, and 350 μmol · m^−2^ · s^−1^ × 1/4NSC induced the highest soluble sugar content. So it was feasible that middle irradiance (250 and 350 μmol · m^−2^ · s^−1^) with 1/4NSC or 3/4NSC could increase the content of soluble protein and sugar, vitamin C, and free amino acid, and reduce nitrate content in lettuce.

### The content of mineral element

**Figure 2:**
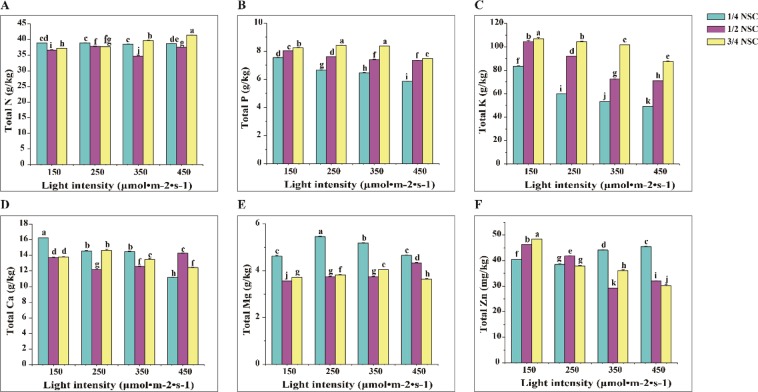
Total N (**A**), P (**B**), K (**C**), Ca (**D**), Mg (**E**) and Zn (**F**) content regulated by different light intensity × NSC. Different letters mark in all figures indicated significant difference (P ≤ 0.05, Tukey’s test). Significant differences among the treatments were determined by SPSS 17.0 for ANOVA.

The light intensity (*p* ≤ 0.01), NSC level (*p* ≤ 0.01) and their combination (*p* ≤ 0.01) exhibited a significant effect on the content of total N, P and K in lettuce (Table S3). The total N content was the highest under 450 μmol · m^−2^ · s^−1^ × 3/4NSC and the lowest under 350 μmol · m^−2^ · s^−1^ × 1/2NSC (Fig. 2A). The content of total P and K in lettuce increased with increasing NSC, while decreased with increasing irradiance, except that the total P under 250 or 350 μmol · m^−2^ · s^−1^ × 3/4NSC was slightly higher than other two light intensity (150 and 450 μmol · m^−2^ · s^−1^) (Fig. 2B,C).

Two-way ANOVA analysis demonstrated that the content of total Ca, Mg and Zn in lettuce were closely associated with light intensity (*p* ≤ 0.01), NSC level (*p* ≤ 0.01) and their interactions (*p* ≤ 0.01) (Table S3). At 1/4NSC, the greatest Ca content was found under irradiance of 150 μmol · m^−2^ · s^−1^ and the least under 450 μmol · m^−2^ · s^−1^, respectively (Fig. 2D). The higher Mg content in lettuce was induced at 1/4NSC under different irradiance condition, with the highest content under 250 μmol · m^−2^ · s^−1^ (Fig. 2E). The lowest Zn content was found under 350 μmol · m^−2^ · s^−1^ × 1/2NSC while the highest content under 150 μmol · m^−2^ · s^−1^ × 3/4NSC (Fig. 2F).

### Antioxidant component content and capacity

**Figure 3:**
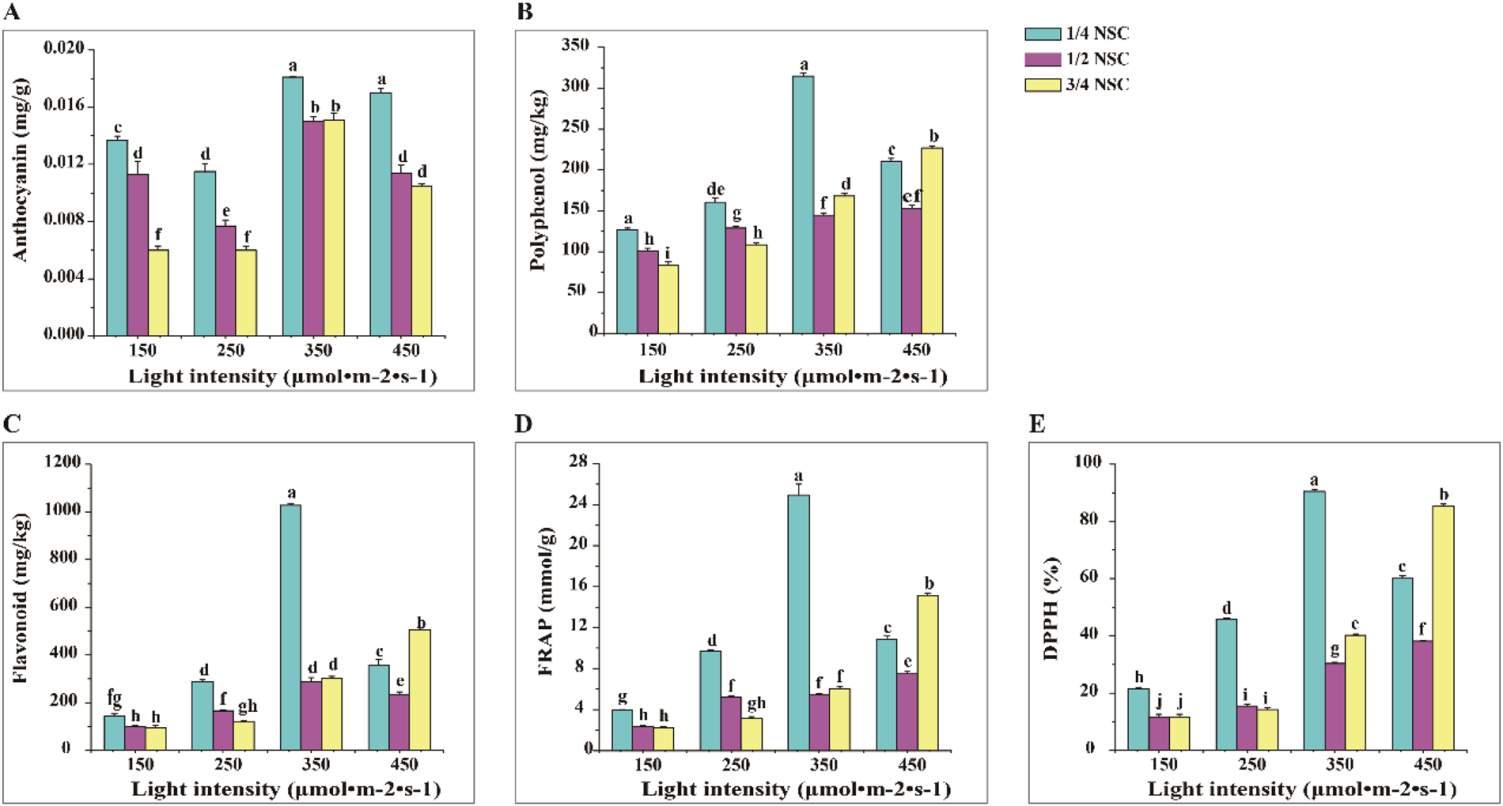
Anthocyanin (**A**), Polyphneol (**B**) and Flavonoid (**C**) content, and FRAP (**D**) and DPPH (**E**) regulated by different light intensity × NSC. Different letters mark in all figures indicated significant difference (P ≤ 0.05, Tukey’s test). Significant differences among the treatments were determined by SPSS 17.0 for ANOVA.

The content of anthocyanin and polyphenol in lettuce was significantly relevant to light intensity (*p* ≤ 0.01), NSC level (*p* ≤ 0.01) and their combination (*p* ≤ 0.01) (Table S4). Anthocyanin content in lettuce significantly decreased with increasing NSC, and the maximal anthocyanin content was shown under 350 μmol · m^−2^ · s^−1^ × 1/4NSC (Fig. 3A). With increasing NSC, the polyphenol content in lettuce decreased under irradiance of 150 μmol · m^−2^ · s^−1^ and 250 μmol · m^−2^ · s^−1^, while those under higher irradiance of 350 and 450 μmol · m^−2^ · s^−1^ decreased at first and then increased (Fig. 3B), the highest polyphenol content was observed in treatment of 350 μmol · m^−2^ · s^−1^ × 1/4NSC (Fig. 3B).

Two-way analysis revealed that flavonoid content, FRAP and DPPH in lettuce were significantly related to light intensity (*p* ≤ 0.01), NSC (*p* ≤ 0.01) and their crosstalk (*p* ≤ 0.01) (Table S4). Under 3/4 NSC, flavonoid content (Fig. 3C), FRAP (Fig. 3D) and DPPH (Fig. 3E) in lettuce markedly increased with increasing irradiance. Flavonoid content, FRAP and DPPH were improved under higher irradiance (350 and 450 μmol · m^−2^ · s^−1^) and the highest flavonoid content, FRAP and DPPH in lettuce were found under 350 μmol · m^−2^ · s^−1^ × 1/4NSC. These meant that 350 μmol · m^−2^ · s^−1^ × 1/4NSC was the most efficient condition for the improvement of antioxidant component content and capacity in lettuce, including anthocyanin, polyphenol, flavonoid, FRAP and DPPH.

To obtain a more detailed understanding of antioxidant role in lettuce under light intensity × NSC, the correlation analysis was performed between antioxidant content and DPPH, FRAP (Table S5). The coefficient of FRAP between contents of polyphenol (r = 0.967, *p* ≤ 0.01), flavonoid (r = 0.970, *p* ≤ 0.01) and DPPH (r = 0.937, *p* ≤ 0.01) were higher than anthocyanin content (r = 0.560, *p* ≤ 0.01). Moreover, the content of anthocyanin, polyphenol and flavonoid were greatly relevant to DPPH (*p* ≤ 0.01), the highest coefficient was found in polyphenol content (r = 0.952), while the minimum in anthocyanin content. Hence, the antioxidant activity mainly derived from polyphenol, flavonoid, anthocyanin in lettuce under light intensity × NSC condition.

## Discussion

Light intensity and nutrient solution significantly affected plant growth and biomass. Plant biomass of tomato increased under 300 and 500 μmol m^−2^ s^−1 ^^8^, while in lettuce increased under 200 μmol · m^−2^ · s^−1 ^^15^. Plant biomass and leaf number of lettuce increased (Table 2) with increasing irradiance from 150 μmol · m^−2^ · s^−1^ to 350 μmol · m^−2^ · s^−1^ as well as with the enhancement of NSC, whereas there might be light stress under 450 μmol · m^−2^ · s^−1^, with decreased biomass and leaf number (Table 2). However, these in lettuce increased under irradiance from 100 μmol · m^−2^ · s^−1^ to 600 μmol · m^−2^ · s^−1^ and decreased under 800 μmol · m^−2^ · s^−1 ^^7^. These results implied that the treatment of 350 μmol · m^−2^ · s^−1^ × 3/4NSC was favourable for lettuce plant growth and biomass.

The lowest irradiance (60 μmol · m^−2^ · s^−1^) combined with higher nitrogen (15 mmol · L^−1^ or 23 mmol · L^−1^) could enhance the content of chlorophyll a and b in lettuce, while the combination of 220 μmo · m^−2^ · s^−1^ × 7 mmol · L^−1^ reduced the content of chlorophyll a and b^15^. In this study, 150 μmol · m^−2^ · s^−1^ × 1/4NSC was conducive to chlorophyll accumulation, whereas the treatment of 450 μmol · m^−2^ · s^−1^ × 1/2NSC was unfavourable for chlorophyll accumulation (Table 3). However, higher chlorophyll content in Chinese kale was induced in the higher fertility treatment^30^. This might be due to that the chlorophyll contents were more affected by light intensity (*p* ≤ 0.01) than NSC (*p* ≤ 0.05) (Table 3). Moreover, the value of chlorophyll a/b under 350 μmol · m^−2^ · s^−1^ × 3/4NSC was higher than other treatment because the content of chlorophyll b drastically reduced (Table 3). Thus, the combination of 350 μmol · m^−2^ · s^−1^ × 3/4NSC in favour of the accumulation of plant biomass might be due to improved photosynthetic capacity of lettuce by increasing of the value chlorophyll a/b.

In plant factory, higher light intensity (250 μmol · m^−2^ · s^−1^ and 300 μmol · m^−2^ · s^−1^) could lead to lower nitrate content but higher content of vitamin C, soluble sugar, soluble protein, and anthocyanin in lettuce^31^. 220–330 μmol · m^−2^ · s^−1^ was the most suitable irradiance level for growth and nutritional quality of *Brassica* microgreens^32^. Similarly, lettuce under higher irradiance of 250 or 350 μmol · m^−2^ · s^−1^ × 1/4NSC had lower nitrate content, and higher contents of soluble protein, vitamin C, soluble sugar and free amino acid (Fig. 1). The nitrate content in lettuce was linearly positively associated with NSC level (Fig. 1B), it was increased linearly in lettuce by increasing N in nutrient solutions^15^, and was lower in medium EC (1.8–2.4) treatment in comparison with high or low EC^33^. It was possible that higher light intensity could promote nitrate accumulation through increasing photosynthetic production^34^. The increase of light intensity could induce accumulation of soluble sugars^9^. In lettuce, soluble sugar content increased under 150 μmol · m^−2^ · s^−1^ to 350 μmol · m^−2^ · s^−1^ and decreased under 450 μmol · m^−2^ · s^−1^, but was negatively regulated by NSC level (Fig. 1D). These indicated that the combination of middle irradiance (250 or 350 μmol · m^−2^ · s^−1^) and 1/4NSC or 3/4NSC could be beneficial to improve nutrition quality in lettuce.

The higher K, Ca and Mg content were observed in ten leafy vegetables under low light intensity (200–400 μmol · m^−2^ · s^−1^) than high intensity (800–1200 μmol · m^−2^ · s^−1^)^11^. In lettuce, total N content was the highest under 450 μmol · m^−2^ · s^−1^ × 3/4NSC while the lowest under 350 μmol · m^−2^ · s^−1^ × 1/2NSC (Fig. 2A). Total P and K content were remarkably enhanced with increasing NSC and decreased with increasing irradiance (Fig. 2B,C), the highest and the least total Ca content were found in lettuce under 150 μmol · m^−2^ · s^−1^ × 1/4NSC and 450 μmol · m^−2^ · s^−1^ × 1/4NSC, respectively (Fig. 2D). The highest total Mg content in lettuce was observed in 250 μmol · m^−2^ · s^−1^ × 1/4NSC treatment (Fig. 2E), and total Zn content in lettuce increased with increasing NSC under 150 μmol · m^−2^ · s^−1^, resulting in the highest content at 150 μmol · m^−2^ · s^−1^ × 3/4NSC (Fig. 2F). K and Ca contents in soybean increased at low light intensity^35^. The content of Ca, Cu, K, Mn and Zn in kale increased under low irradiance (125–335 μmol · m^−2^ · s^−1^) but the P content decreased^10^. However, the mineral contents in spinach were significant different under different irradiance levels, content of Ca and Fe decreased at low light levels^10^. Mineral nutrient played a crucial role in photosynthesis, carbohydrate content in plant^36^. P, K, Ca, Mg, Zn in lettuce mainly were accumulated under higher light intensity (350 and 450 μmol · m^−2^ · s^−1^) × 1/4 or 3/4NSC (Fig. 2). These might be favor to the highest lettuce biomass in 350 μmol · m^−2^ · s^−1^ × 3/4NSC.

Phytochemicals, including anthocyanin, polyphenol, flavonoid, played a wide range of therapeutic and health-promoting role for human body. The content of anthocyanin, polyphenol, flavonoid, and FRAP, DPPH in lettuce were significantly affected by light intensity (*p* ≤ 0.01), NSC level (*p* ≤ 0.01) and their combination (*p* ≤ 0.01) (Table S4). These were lower under lower irradiance (150 and 250 μmol · m^−2^ · s^−1^) than higher irradiance (350 and 450 μmol · m^−2^ · s^−1^), the highest contents were exhibited under 350 μmol · m^−2^ · s^−1^ × 1/4NSC (Fig. 3). Compare with high intensity (800–1200 μmol · m^−2^ · s^−1^), low light intensity (200–400 μmol · m^−2^ · s^−1^) could improve antioxidant activity for ten leafy vegetables^15^. *Brassica* microgreens possessed the highest anthocyanin content under 330–440 μmol · m^−2^ · s^−1^ irradiance^32^. Anthocyanin content in lettuce decreased with increasing NSC level (Fig. 3A), while increased in radishes under lower solution concentration^37^. Polyphenol content in vegetable could increase under higher light intensity, and also was affected by solution concentration^37^. Polyphenol content in lettuce decreased with increasing NSC under lower irradiance (150 and 250 μmol · m^−2^ · s^−1^), and decreased at first and then increased under higher irradiance (350 and 450 μmol · m^−2^ · s^−1^) (Fig. 3B). Plant in response to the lower fertilizer concentrations induced the increasing content of polyphenol and flavonoid^38^. Generally, the highest contents of anthocyanin, polyphenol, flavonoid, FRAP and DPPH were observed at the lowest solution concentration (Fig. 3). Flavonoid content, FRAP and DPPH were the highest under 350 μmol · m^−2^ · s^−1^ × 1/4NSC (Fig. 3). These indicated that the antioxidant content and capacity in lettuce, including anthocyanin, polyphenol, flavonoid, FRAP and DPPH, were most improved under 350 μmol · m^−2^ · s^−1^ × 1/4NSC. There was significant coefficient between polyphenol (r = 0.967, *p* ≤ 0.01), flavonoid (r = 0.970, *p* ≤ 0.01), DDPH (r = 0.937, *p* ≤ 0.01), anthocyanin (r = 0.560, *p* ≤ 0.01) and FRAP (Table S5). Thus, polyphenol, flavonoid, DPPH and anthocyanin mainly played antioxidant roles in lettuce under light intensity × NSC condition.

## Conclusion

In plant factory, light and nutrient solution are the most effective factors improving yield and quality of vegetable. This study clearly demonstrated that the combination of light intensity and nutrient solution could significantly affect growth and quality of lettuce. The interaction of 350 μmol · m^−2^ · s^−1^ × 3/4NSC or 1/4NSC was conducive to growth of lettuce, while the irradiance of 250 and 350 μmol · m^−2^ · s^−1^ × 1/4 or 3/4NSC contributed to increased content of soluble protein and sugar, vitamin C, nitrate and free acid, and 350 μmol · m^−2^ · s^−1^ × 1/4NSC exhibited a dramatically effect on improving antioxidant content and capacity. The 350 μmol · m^−2^ · s^−1^ × 1/4NSC treatment was the more suitable condition for lettuce production in plant factory.

## Supplementary information

Supplementary Information.
