# Supplementary material for: Nutritional quality, mineral and antioxidant content in lettuce affected by interaction of light intensity and nutrient solution concentration
Source: Sci Rep. 2020 Feb 18;10:2796. doi: 10.1038/s41598-020-59574-3 (PMC7029024; doi:10.1038/s41598-020-59574-3)
Supplement: Supplementary file 1 — Supplementary Information. [file 41598_2020_59574_MOESM1_ESM.pdf]

Nutritional quality, mineral and antioxidant content in lettuce affected by interaction of light intensity and nutrient solution concentration

Jiali Song[1], Hui Huang[1], Yanwei Hao[1], Shiwei Song[1], Yiting Zhang[1], Wei Su[1] and Houcheng Liu[1*]

College of Horticulture, South China Agricultural University, Guangzhou 510642, China;
Song_46@126.com; 812991303@qq.com; yanweihao@scau.edu.cn; swsong@scau.edu.cn;
yitingzhang@scau.edu.cn; susan_l@scau.edu.cn;
* Correspondence: liuhch@scau.edu.cn; Tel.: +020-85280464

Table S1. The concentration of mineral element in different strength nutrient solution concentration (NSC)

NSC \ Mineral nutrition (mg·L-1)	N	P	K	Ca	Mg	S	Fe	B	Mn	Zn	Cu	Mo
**The full-strength**	210	31	234	160	48	64	5.6	0.5	0.5	0.05	0.02	0.01
**1/4**	52.5	7.75	58.5	40	12	16	1.4	0.125	0.125	0.0125	0.005	0.0025
**1/2**	105	15.5	117	80	24	32	2.8	0.25	0.25	0.025	0.01	0.005
**3/4**	157.5	23.25	175.5	120	36	48	4.2	0.375	0.375	0.0375	0.015	0.0075

Table S2. Two-way ANOVA analysis of nutritional quality accumulated under different light intensity×NSC

**Variable**	**Light intensity**	**NSC**	**Light intensity×NSC**
Soluble protein	236.214**	44.522**	19.709**
Nitrate	64.027**	374.536**	48.163**
Vitamin C	23.489**	38.536**	19.189**
Soluble sugar	715.27**	306.197**	30.773**
Free amino acid	323.935**	33.439**	8.497**

* and ** represented the significant difference at $p \leq 0.05$ and $p \leq 0.01$, respectively. Significant differences among the treatments were determined by SPSS 17.0 for ANOVA. The two-way ANOVA was used the interactive type.

Table S3. Two-way ANOVA analysis of mineral element accumulated under different light intensity × NSC.

**Variable (Total)**	**Light intensity**	**NSC**	**Light intensity×NSC**
N	207.106**	789.983**	244.179**
P	1275.855**	5163.293**	241.042**
K	2484.291**	7925.417**	243.715**
Ca	413.887**	183.763**	463.742**
Mg	270.927**	5263.155**	329.996**
Zn	1184.811**	601.914**	905.772**

* and ** represented the significant difference at $p \le 0.05$ and $p \le 0.01$, respectively. Significant differences among the treatments were determined by SPSS 17.0 for ANOVA. The two-way ANOVA was used the interactive type.

Table S4. Two-way ANOVA analysis of antioxidant component accumulated under different light intensity × NSC.

**Variable**	**Light intensity**	**NSC**	**Light intensity×NSC**
Anthocyanin	171.248**	172.054**	10.067**
Polyphenol	636.294**	466.725**	152.21**
Flavonoid	762.647**	513.346**	313.33**
FRAP	487.726**	491.057**	257.973**
DPPH	3502.69**	2221.999**	800.203**

* and ** represented the significant difference at $p \le 0.05$ and $p \le 0.01$, respectively. Significant differences among the treatments were determined by SPSS 17.0 for ANOVA. The two-way ANOVA was used the interactive type.

Table S5. The correlation analysis of antioxidant component and capacity affected by light intensity × NSC.

**Parameter**	**Polyphenol**	**Flavonoid**	**Anthocyanin**	**DPPH**	**FRAP**
Polyphenol	1				
Flavonoid	0.958**				
Anthocyanin	0.677**	0.615**			
DPPH	0.952**	0.887**	0.591**		
FRAP	0.967**	0.970**	0.560**	0.937**	1

* and ** represented the significant difference at $p \le 0.05$ and $p \le 0.01$, respectively. Significant differences were determined by SPSS 17.0.

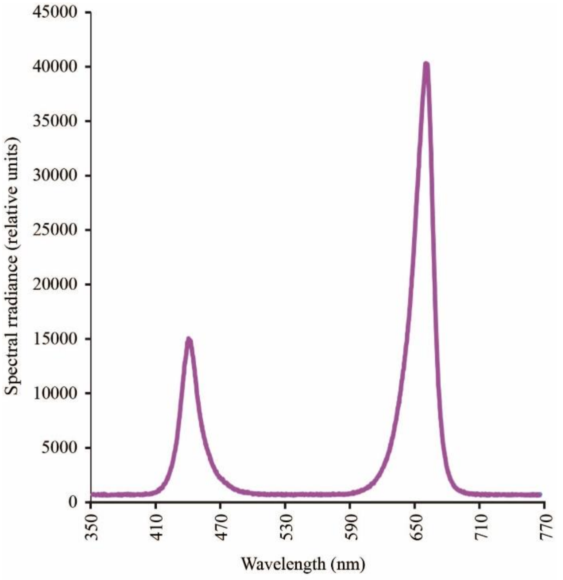

Figure S1. The Spectral distributions of LEDs in this study.

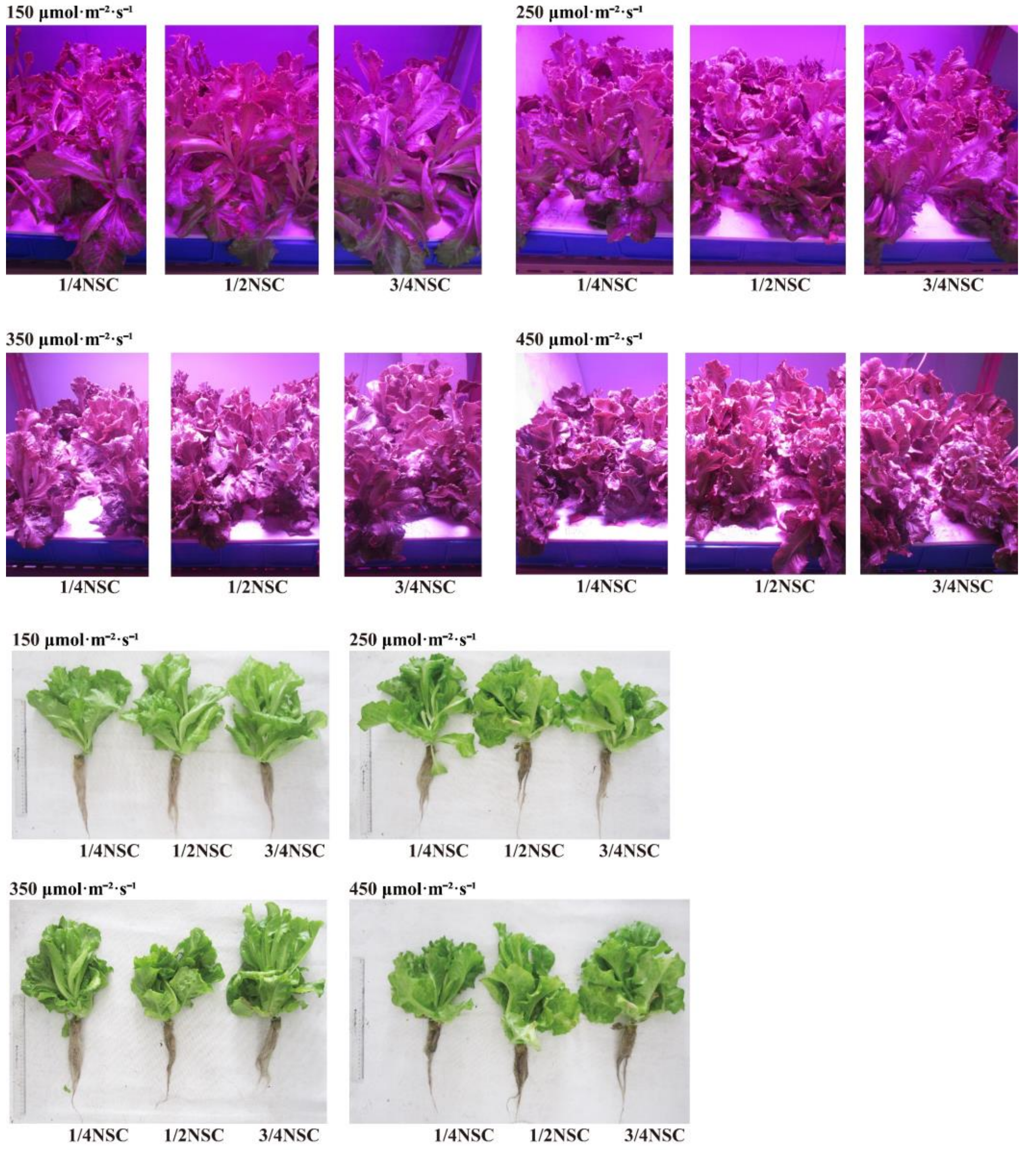

Figure S2. The phenotype of lettuce plant under different light intensity×NSC.
